# The longitudinal effects of seated isometric yoga on blood biomarkers, autonomic functions, and psychological parameters of patients with chronic fatigue syndrome: a pilot study

**DOI:** 10.1186/s13030-019-0168-x

**Published:** 2019-11-05

**Authors:** Takakazu Oka, Tokusei Tanahashi, Battuvshin Lkhagvasuren, Yu Yamada

**Affiliations:** 1 Department of Psychosomatic Medicine, Graduate School of Medical Sciences, Kyushu University, Fukuoka, 812-8582 Japan; 2 Department of Psychosomatic Medicine, International University of Health and Welfare Hospital, Iguchi 537-3, Nasushiobara-shi, Tochigi-ken, 329-2763 Japan; 3 Brain Science Institute, Mongolian National University of Medical Sciences, Zorig Street 3, Ulaanbaatar, 14210 Mongolia

**Keywords:** Chronic fatigue syndrome, Isometric yoga, Myalgic encephalomyelitis, TNF-α, Alexithymia, Cytokine, Heart rate variability

## Abstract

**Background:**

In a previous randomized controlled trial, we found that practicing seated isometric yoga regularly for 2 months improved the fatigue of patients with chronic fatigue syndrome (CFS) who are resistant to conventional therapy. The aim of this pilot study was to investigate the possible mechanisms behind this finding by comparing blood biomarkers, autonomic nervous function, and psychological indices before versus after an intervention period of seated isometric yoga practice.

**Methods:**

Fifteen patients with CFS who did not show satisfactory improvements after at least 6 months of conventional therapy practiced seated isometric yoga (biweekly 20-min sessions with a yoga instructor and daily practice at home) for 2 months. The longitudinal effects of seated isometric yoga on fatigue, blood biomarkers, autonomic function, and psychological state were investigated by comparing the following parameters before and after the intervention period: Fatigue severity was assessed by the Chalder fatigue scale (FS) score. Levels of the blood biomarkers cortisol, DHEA-S, TNF-α, IL-6, prolactin, carnitine, TGF-β1, BDNF, MHPG, HVA, and α-MSH were measured. The autonomic nervous functions assessed were heart rate (HR) and HR variability. Psychological indices included the 20-item Toronto Alexithymia Scale (TAS-20) and the Hospital Anxiety and Depression Scale (HADS).

**Results:**

Practicing seated isometric yoga for 2 months resulted in significant reductions in the Chalder FS (*P* = 0.002) and HADS-depression (*P* = 0.02) scores. No significant changes were observed in any other parameter evaluated. The change in Chalder FS score was not correlated with the change in HADS-depression score. However, this change was positively correlated with changes in the serum TNF-α levels (*P* = 0.048), the high frequency component of HR variability (*P* = 0.042), and TAS-20 scores (*P* = 0.001).

**Conclusions:**

Regular practice of seated isometric yoga for 2 months reduced the fatigue and depressive symptom scores of patients with CFS without affecting any other parameters we investigated. This study failed to identify the markers responsible for the longitudinal fatigue-relieving effect of seated isometric yoga. However, considering that the reduced fatigue was associated with decreased serum TNF-α level and TAS-20 scores, fatigue improvement might be related to reduced inflammation and improved alexithymia in these patients.

**Trial registration:**

University Hospital Medical Information Network (UMIN CTR) UMIN000009646. Registered Dec 27, 2012.

## Background

Chronic fatigue syndrome (CFS), or myalgic encephalomyelitis (ME), is a debilitating disease characterized by persistent or relapsing unexplained fatigue lasting at least 6 months that is not relieved by rest and that causes a substantial reduction in daily activities [1, 2].

The pathophysiological mechanisms that cause fatigue in CFS are not fully understood. However, previous studies have suggested that patients with CFS display several abnormalities and/or alterations, including those of the hypothalamic–pituitary–adrenocortical (HPA) axis, immune system, central nervous system, autonomic nervous system (ANS) and mitochondrial energy metabolism [3]. HPA axis dysfunctions include attenuated diurnal changes in the cortisol level [4, 5] and a decreased blood level of dehydroepiandrosterone sulfate (DHEA-S) [6, 7]. The immune system abnormalities include increased blood levels of transforming growth factor (TGF)-β1 and several proinflammatory cytokines such as tumor necrosis factor (TNF)-α and interleukin (IL)-6 [8–11]. Dysfunctions in the central nervous system include hypoactivity of the dopaminergic nervous system in response to reward stimuli [12, 13]. It has also been reported that the plasma levels of 3-methoxy-4-hydroxyphenylglycol (MHPG), a major metabolite of noradrenaline that reflects noradrenergic neural tone [14, 15], are lower [16] and α-melanocyte stimulating hormone (α-MSH) levels are higher than in healthy subjects [17]. Autonomic dysfunctions include a high baseline heart rate (HR) [18–20], low vagal tone and sympathetic overactivity as assessed by HR variability (HRV) [19, 20]. Mitochondrial energy metabolism abnormalities include low-level acylcarnitine [21, 22]. Furthermore, patients with CFS sometimes have comorbid depressive disorders [23] and characteristics of alexithymia, which is characterized by difficulty identifying and expressing emotions [24].

In a randomized controlled trial (RCT), we observed that regular practice of seated isometric yoga for 2 months reduced fatigue, as assessed by the Chalder fatigue scale (FS) score, and improved the general health perception of patients with CFS who were resistant to conventional therapy [25]. Furthermore, we also found that even a single session of practicing seated isometric yoga could reduce fatigued mood (as assessed by the Profile of Mood States fatigue score) when patients became accustomed to the procedures and could practice them successfully [25].

However, the mechanism by which seated isometric yoga improves the fatigue of patients with CFS remains unknown. Given that specific biomarkers of CFS have yet to be identified, it is impossible to determine if practicing seated isometric yoga can attenuate or counteract the causal mechanisms of the disease. However, we hypothesized that practicing seated isometric yoga could help normalize at least some of the abnormalities or alterations reported for patients with CFS, and these changes might be associated with the fatigue-relieving effects of seated isometric yoga.

Therefore, we first assessed the effect of a single session of seated isometric yoga (short-term effect) on several blood biomarkers and autonomic function. We found that a single session of seated isometric yoga with a yoga instructor resulted in beneficial changes, i.e. increases in the serum DHEA-S level and the high frequency (HF) component of HRV, and decreases in HR, serum cortisol, and TNF-α levels. All of these changes shifted the abnormal parameters of the CFS patients closer to those of healthy subjects [26].

As the next step in this study, to assess the longitudinal effects, we sought to investigate the effects of regular seated isometric yoga practice for 2 months on the abnormalities of patients with CFS. If the longitudinal benefits could be obtained exclusively by the repetition or accumulation of short-term effects, regular practice of seated isometric yoga should influence the same parameters as those affected by short-term practice. However, alternatively, it is also possible that the short-term and extended fatigue-relieving effects of seated isometric yoga are associated with different mechanisms. That is, the short-term effects may be associated with changes in autonomic and neurotransmitter functions within the brain and reduction of stress markers, all of which can show quantifiable differences within 1 h. In contrast, the longitudinal effects might be associated with changes in immune function, energy metabolism, or changes in psychological and behavioral factors, which require longer periods to change, in addition to the cumulative benefits of the short-term effects [26].

This hypothesis is also based on findings from studies that investigated the effects of yoga in breast cancer patients or survivors. Although those specific programs were different from that of present study, regular yoga practice resulted in recovery of the disrupted diurnal rhythm of cortisol secretion [27], reduced proinflammatory markers such as TNF-α [28, 29], and reduced the fatigue [27, 30] of these patients. Furthermore, regular yoga practice showed numerous psychological benefits, such as reduction of perceived stress, anxiety, and depression (for review, see [31]). Therefore, it is of interest to know if regular practice of seated isometric yoga can also provide such psychological benefits. Thus, we hypothesized that regular practice of seated isometric yoga not only has the same effects on blood-based biomarkers and autonomic function as short-term practice, but also elicits further psychological benefits.

To test this hypothesis and to begin to obtain insights into the mechanisms underlying the longitudinal, fatigue-relieving effects of seated isometric yoga, we evaluated changes in blood biomarker levels, autonomic function, and psychological parameters before versus after a period of yoga intervention (regular practice of seated isometric yoga for approximately 2 months) and assessed the correlations between reduced fatigue and the changes in these parameters. This is the first study to demonstrate the longitudinal effects of seated isometric yoga in patients with CFS.

## Methods

### Design

**Fig. 1:**
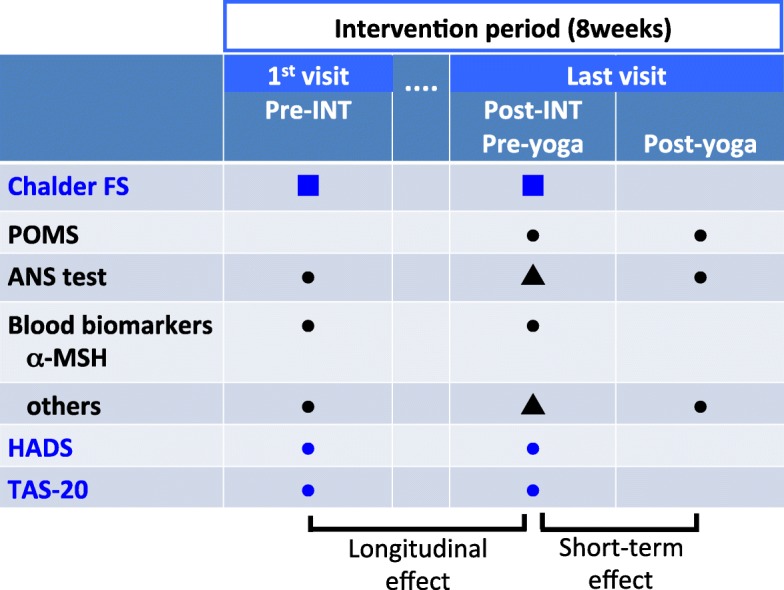
Schematic representation of this study and related previous studies. In the first study, we compared the changes in Chalder FS scores in yoga and control groups in an RCT [25]. In the second study, we investigated the short-term effect of seated isometric yoga, in which POMS, ANS function, and blood biomarkers except α-MSH were compared just before (pre-yoga) and after a single session of seated isometric yoga (post-yoga) on the day of the last visit of the intervention period [26]. In this study, we investigated the longitudinal effects of seated isometric yoga, in which Chalder FS scores, ANS function, blood biomarkers, HADS, and TAS-20 were compared on the day of the first visit before practicing yoga (pre-intervention) and the day of the last visit before practicing yoga (post-intervention) following an intervention period. Marks in blue were measured in both the yoga group and the control group. Marks in black were measured only in the yoga group. In this study, we again showed changes in the Chalder FS scores (■), which had been previously reported [25]. Most, but not all, data on the blood biomarkers and ANS tests of the pre-yoga period in the second study [26] were the same as those of the post-intervention period in the present study. For details, please read the associated text. INT, intervention

Analysis was based on our RCT that was published previously [25]. The designs of this study and related previous studies are illustrated in Fig. 1. In the first study, we compared changes in the Chalder FS scores between subjects who were treated with conventional pharmacotherapy (control group) and subjects who practiced seated isometric yoga together with conventional pharmacotherapy (yoga group). We also compared the Profile of Mood States fatigue scores just before and after a single session of seated isometric yoga with an instructor and on the final day of practicing yoga with an instructor [25]. In the second study, to understand the possible mechanisms underlying the acute fatigue-relieving effects of yoga, we compared the results of ANS functional tests and blood biomarkers before and after participating in a single session of seated isometric yoga with an instructor and on their last day [26]. In the present study, we compared the Chalder FS scores, ANS function tests, blood biomarkers, and psychological parameters between the first day (pre-intervention) and the pre-yoga practice results on their last day (post-intervention). Thus, the present study is the second in which we show the changes in the Chalder FS score; the first was reported previously [25]. Furthermore, we utilized the values of most blood biomarkers and ANS function tests from the pre-yoga period in the previous study [26] as those of the post-intervention period in the present study. Specifically, the plasma values and those of other parameters, such as IL-6, PRL, and carnitine, were the same. However, due to the lack of data for some values from the pre-intervention and post-yoga time points, matched pairs for some parameters of ANS function and serum biomarkers were not exactly the same between this study and the previous one [26] (this comprised only one or two values among the data from the 15 participants).

All aspects of this study were approved by the Institutional Review Board of Kyushu University (approval number 27071), and it was pre-registered in the University Hospital Medical Information Network (registration number: UMIN000009646). Written informed consent was obtained from all participants before they were enrolled.

### Subjects

The study included patients with CFS who were enrolled in the yoga group (*n* = 15) and control group (*n* = 15) in our previous RCT [25]. The diagnosis of CFS was made based on the diagnostic criteria of the 1994 CFS research case definition [1]. Furthermore, diagnoses also met the 2011 international consensus criteria for ME [23] and the 2015 diagnostic criteria for systemic exertion intolerance disease [32]. The inclusion criteria for this study were as follows: (1) no significant improvement in fatigue despite at least 6 months of conventional treatment at our university hospital [33–35], (2) aged 20–70 years old, (3) severity of fatigue causing an absence from school or the workplace for at least several days per month but not so severe as to require assistance with activities of daily living, (4) ability to fill out questionnaires without assistance, (5) ability to sit for at least 30 min and (6) ability to visit the hospital every 2 or 3 weeks.

### Procedures

Assessments were made pre- and post-intervention, i.e. on the first and last day, respectively, of the 2-month seated isometric yoga intervention conducted by a yoga instructor. On both days, assessments were conducted just before participating in the yoga session. On the day of assessment, patients in the yoga group filled out self-rating questionnaires and underwent autonomic function testing, blood sampling, and a medical check-up by their doctors-in-charge before practicing yoga. The questionnaires were collected by a nurse who was not involved in the study. Blood samples were drawn from the forearm veins between 2 p.m. and 4 p.m., centrifuged, and stored at − 80 °C until the assays were performed. For some patients, it was difficult to draw a sufficient volume of blood to measure all biomarkers. Therefore, we had to eliminate the assessment of several biomarkers for some participants (see the results section). In contrast, patients in the control group only filled out self-rating questionnaires and did not have blood drawn or do autonomic function tests. This was partly to minimize their physical discomfort, as some patients showed evident allodynia and even the placement of the sphygmomanometer cuff caused substantial pain.

During the intervention period, any medications that had been prescribed previously as part of conventional pharmacotherapy were continued at the same dosage. These drugs included antidepressants such as duloxetine, hypnotics, pregabalin, coenzyme Q10, or Japanese traditional herbal medicines such as hochuekkito, which is often prescribed to improve energy for (Qi)-deficient conditions [34, 36]. No patient was treated with hydrocortisone, non-steroidal anti-inflammatory drugs, or drugs that affect the dopaminergic system such as sulpiride.

### Yoga intervention

Participants practiced seated isometric yoga, consisting of biweekly 20-min sessions with a yoga instructor and daily in-home sessions, for approximately 2 months (for a video of this program, see [37]). They practiced seated isometric yoga an average of 5.8 times per week, which was determined based on the yoga diaries maintained by the patients [25]. Seated isometric yoga was practiced in a quiet room on a one-to-one basis with a yoga instructor. The instructor had more than 30 years of experience as a yoga instructor and had more than 4 years of experience instructing patients with CFS [26]. The precise seated isometric yoga routine has been described elsewhere [25]. In brief, the program consists of three parts. First, patients practice being aware of their spontaneous breathing while placing the right hand on the lower abdomen and the left hand on the back for 1 min to facilitate interoceptive and proprioceptive awareness. Second, patients practice six isometric poses four to six times, then two to three times while making sounds like “Uh…” or “Ah….”, followed by two to three times without making sounds. Isometric postures were practiced slowly, taking 3–10 s (average: 5 s) for each in association with exhalation using 50% of maximal physical strength. Tension was then released slowly with inhalation. Then, the hands and body returned slowly to the basic seated position, taking 3–10 s along with exhalation with/without sounds. The length and number of repetitions varied depending on the severity of fatigue and psychological and physiological tension of the patient. However, the patients were encouraged to synchronize their motions with breathing. Third, the patients observed naturally arising abdominal breathing for 1 min.

### Measures

#### Fatigue

The severity of fatigue was assessed using the Chalder FS, which is a well-validated self-reported scale that measures the physical and mental symptoms of fatigue [38, 39]. In this study, we used the 14-item version of the Chalder FS, in which the highest score possible is 42.

#### Blood biomarkers

##### Serum markers

Serum DHEA-S levels were measured by chemiluminescent enzyme immunoassay and cortisol and PRL levels by an electrochemiluminescence immunoassay. TNF-α levels were measured by an enzyme-linked immunosorbent assay (ELISA) using the Quantikine high-sensitivity ELISA human TNF-α immunoassay (R&D Systems, Inc., Minneapolis, MN, USA), with a minimum detectable concentration of 0.07 pg/mL. IL-6 levels were measured using a human IL-6 chemiluminescent enzyme immunoassay cartridge (Fujirebio, Tokyo, Japan), with a minimum detectable concentration of 0.2 pg/mL. Total carnitine, acylcarnitine and free carnitine concentrations were measured using the enzyme cycling method.

##### Plasma markers

TGF-β1 and brain-derived neurotrophic factor (BDNF) levels were measured in plasma using Quantikine ELISA human kits (R&D Systems, Inc.) specific for TGF-β1 and BDNF, with minimum detectable concentrations of 0.50 and 20 pg/mL, respectively. Plasma levels of MHPG and homovanillic acid (HVA) were measured by high-performance liquid chromatography. α-MSH levels were measured by radioimmunoassay (Euro Diagnostica, Malmo, Sweden).

#### Autonomic nervous function

Non-invasive testing of ANS function was conducted by analyzing HRV. After a sufficient resting period, patients underwent a 3-lead electrocardiogram for 2 min while in the seated position. Electrodes were placed on both wrists. Beat-to-beat HR was assessed on the electrocardiogram, and HRV indices and respiratory rate were measured using the software program Kiritsu Meijin (Crosswell Co., Inc., Yokohama, Japan) [40] and the HR monitor LRR-03 (GMS, Tokyo, Japan). The majority of previous studies have used spectral techniques based on the fast Fourier transform algorithm, which is insufficient to estimate the precise power spectral density from short time series data. Therefore, in Kiritsu Meijin, HRV was analyzed using the MemCalc method [41], which is a new technique for time series analyses. It involves a combination of maximum entropy spectral analysis and non-linear least squares fitting. This enabled reliable analyses of the low frequency (LF; 0.05–0.15 Hz) and HF (HF; 0.15–0.4 Hz) components over a minimum interval of 30 s. Time domain analysis and spectral analyses of HRV using the MemCalc system were performed over a 1-min period. In the time domain analysis, the coefficient of variation of R-R intervals (CV_R-R_) was determined. HF was used as an index of parasympathetic activity and LF as an index of both sympathetic and parasympathetic activities. LF/HF was used as an index of sympathetic activity [42, 43].

#### Psychological parameters

We administered two self-rating questionnaires to assess the changes in psychological indices, i.e. anxiety, depression, and alexithymia. To assess anxiety and depression, the Japanese version of the Hospital Anxiety and Depression Scale (HADS) [44, 45] was used. The HADS contains two subscales: the anxiety (HADS-A) and depression (HADS-D) subscales. To assess alexithymia, the Japanese version of the 20-item Toronto Alexithymia Scale (TAS-20) [46] was used. The HADS and TAS-20 were administered to both the yoga group and control group.

### Statistical analyses

The normality of the data distribution was evaluated by the Kolmogorov–Smirnov test. Differences in the Chalder FS, TAS-20, HADS-A, and HADS-D scores were tested by two-way, repeated measures, analysis of variance (ANOVA) of the mean scores. Two comparisons were made: one compared the scores of the yoga group to those of the control group (between-subject effect); the other compared the scores measured before the intervention to those measured after (within-subject effect). Effect sizes for significant changes in the self-reported psychological questionnaires were calculated using Cohen’s d with a 95% confidence interval. Effect sizes of 0.20 were considered small, 0.50 moderate, and 0.80 large [47]. Differences in the plasma/serum biomarker levels and autonomic function indices and psychological test scores before and after the yoga intervention were analyzed using a paired-sample *t* test, or the Wilcoxon signed-rank test if the Kolmogorov–Smirnov test yielded significant results. To avoid the accumulation of type 1 error across the eight serum biomarkers, five plasma biomarkers, and six autonomic function indices, *P* values have been adjusted using an adaptive linear step-up procedure that controls for a false discovery rate (FDR). The FDR control at 5% was calculated separately for the parameters (e.g., serum biomarkers, plasma biomarkers, and autonomic function indices) to allow for group differences in the parameters before and after the intervention of the null hypotheses [48]. Correlations between the Chalder FS score and plasma/serum biomarker levels, autonomic function indices, and psychological test scores were identified using bivariate Pearson’s correlation analysis or the Spearman’s correlation analysis if the Kolmogorov–Smirnov test yielded significant results. Correlated variables (as independent variables) were further analyzed by multiple linear regression analysis to determine their causal relationships with the Chalder FS score (as the dependent variable), using pairwise deletion for missing values. According to a post-hoc power analysis using G*Power software, v3.1.7 [49], the power achieved for the paired-sample, two-tailed *t* test of the 15 subjects was computed as 98%. Statistical significance was set at *p* < 0.05, and all tests were two-tailed. Data are presented as means ± standard deviation with 95% confidence intervals (Table 1). Data were analyzed using SPSS for Windows, v21.

**Table 1:** Changes in Chalder FS scores and blood biomarkers by a two-month yoga intervention in the yoga group

Profile	RV	Pre	Post	∆	95% CI	UPV	FPV	C	FC
Fatigue									
Chalder FS score		25.9 ± 6.1	19.2 ± 7.5	−6.7 ± 6.9	− 10.6; − 2.9	**0.002**	**0.002**		
Serum biomarkers									
Cortisol (μg/dL)	3–20	11.5 ± 8.3	*10.4 ± 6.3*	−1.0 ± 4.7	−3.7; 1.7	0.552	1	0.601	0.801
DHEA-S (μg/dL)	13–290	178.4 ± 67.7	180.5 ± 66.5	2.1 ± 25.2	−12.5; 16.6	0.763	1	0.794	0.907
TNF-α (pg/mL)	0.7–1.7	*0.9 ± 0.2*	0.8 ± 0.2	−0.1 ± 0.3	− 0.2; 0.1	0.478	1	**0.006**	**0.048**
IL-6 (pg/mL)	< 4.0	*1.1 ± 0.6*	1.2 ± 0.6	0.1 ± 0.7	−0.3; 0.5	0.478	1	0.833	0.833
PRL (ng/mL)	< 20	11.4 ± 6.3	11.1 ± 6.3	*−0.3 ± 5.9*	−3.6; 2.9	0.839	0.958	0.219	0.876
Carnitine									
Total (μmol/L)	45–91	53 ± 12.5	52.6 ± 12.4	0.4 ± 10.0	−5.9; 5.2	0.885	0.885	0.292	0.584
Free (μmol/L)	36–74	43.9 ± 11.3	43.1 ± 11.2	−0.8 ± 10.3	−6.5; 4.9	0.767	1	0.538	0.861
Acyl (μmol/L)	6–23	9.1 ± 3.1	9.6 ± 3.7	0.4 ± 3.4	−1.5; 2.3	0.650	1	0.240	0.639
Plasma biomarkers									
TGF-β_1_(ng/mL)	1.6–3.2	*13.1 ± 16.2*	10 ± 8.5	*−3.1 ± 14.1*	−11.6; 5.5	0.753	0.941	0.943	0.943
BDNF (pg/mL)	6186–42,580	*5758 ± 8184*	*4237 ± 4522*	− 1521 ± 5442	− 4663; 1621	0.594	0.990	0.922	1
MHPG (ng/mL)	3.2–5.9	3.6 ± 1.1	*3.7 ± 1.3*	0.1 ± 0.8	−0.3; 0.5	0.925	0.925	0.880	1
HVA (ng/mL)	4.4–15.1	9.1 ± 3.7	*10.9 ± 9.4*	*1.8 ± 6.7*	−1.9; 5.5	0.379	0.947	0.257	1
α-MSH (pg/ml)	12.7–14.5	16.8 ± 5.2	17.6 ± 5.1	0.8 ± 1.5	−0.2; 1.8	0.112	0.560	0.580	1
Autonomic function indices									
HR (bpm)		88.1 ± 8.5	85 ± 9.5	−3.1 ± 12.0	−10.3; − 4.1	0.369	1	0.096	0.288
HF (ms^2^)	83–3630	74.7 ± 79.4	70.4 ± 62.3	−4.3 ± 87.4	−57.1; 48.5	0.861	1	**0.007**	**0.042**
LF (ms^2^)	193–1009	*163.4 ± 197.1*	*204.9 ± 363.9*	*41.5 ± 434.3*	− 220.9; 304.0	0.972	0.972	0.563	0.563
LF/HF	1.1–11.6	*4.5 ± 5.4*	*4.8 ± 7.1*	*−0.2 ± 5.1*	−2.8; 3.3	0.463	1	0.341	0.682
CV_R-R_ (%)		*3.5 ± 1.6*	3.5 ± 1.5	−0.1 ± 2.3	−1.4; 1.3	0.507	0.760	0.351	0.526
RR (/min)		17.4 ± 3.9	16.4 ± 5.2	−1.0 ± 5.1	−4.1; 5.1	0.497	0.994	0.538	0.645

Values are mean ± standard deviation

*RV* Reference values

*Pre* Mean values before intervention

*Post* Mean values after intervention

∆: The mean difference between Pre and Post values, i.e. Post value – Pre value

*P value* Paired *t*-test or Wilcoxon signed-rank test between Pre and Post values

*95% CI* 95% Confidence interval of ∆ (Lower bound; Upper bound)

*UPV* Unadjusted *P* values

*FPV* FDR-adjusted *P* values for multiple comparisons

*C* Correlation between ∆Chalder FS scores and ∆ values of plasma/serum biomarkers and autonomic function indices; two-tailed

*FC* FDR-adjusted *P* values for the correlations

*n.s* not significant, *n* = 11–15

The numbers on Italic: The probability of normal distribution is violated by Kolmogorov-Smirnov test

The numbers on bold indicate there is a significant difference

## Results

This study comprised 15 participants in the yoga group (age range: 24–60 years; mean age [mean ± SD]: 38.0 ± 11.1 years; 3 men and 12 women) and 15 in the control group (age range: 20–59 years; mean age: 39.1 ± 14.2 years; 3 men and 12 women) from a previous study [25]. We compared changes in Chalder FS scores and psychological parameters in both the yoga group and the control group. However, we compared blood biomarkers and autonomic function parameters only in the yoga group. This was partly due our attempt to minimize the discomfort of patients who showed evident allodynia, for whom even wrapping the sphygmomanometer cuff can be painful. In the yoga group, we were unable to determine the DHEA-S, TNF-α, IL-6, BDNF, and MHPG levels of one patient, the cortisol and TGF-β1 levels of two patients, and the α-MSH level of four patients due to a shortage of blood for analysis. We could not obtain autonomic function parameters on the first day of the intervention period from two patients due to noise caused by muscular movements. The missing data were excluded by pairwise deletion during data processing. The details of the sample data are described in Tables 1 and 2.

**Table 2:** Effects of a two-month intervention on psychological test scores of the patients of the yoga group and those of the control group (each group *n* = 15)

Profile	∆		Independent-sample *t* test (*P*)	2-way repeated ANOVA (*P*)			Effect Size between Groups (Cohen’s *d*)
	Yoga group	Control group		Group	Time	Group x Time	
Chalder FS	−6.7 ± 6.9	−0.3 ± 3.4	0.004	0.124	0.001	0.003	−1.165
TAS-20	−0.4 ± 5.9	−0.3 ± 5.7	0.950	0.778	0.755	0.950	−0.017
HADS-A	−0.8 ± 2.4	−0.5 ± 2.9	0.786	0.172	0.181	0.786	−0.113
HADS-D	−2.6 ± 3.8	0.9 ± 2.7	0.008	0.075	0.163	0.008	−1.062

Values are mean ± standard deviation

∆: The mean difference between Pre and Post values, i.e. Post value – Pre value

*P* values Tested with two-way repeated measures analysis of variance (ANOVA) and unpaired-sample *t*-test

### Longitudinal effects of yoga on fatigue, blood biomarker levels, and ANS function

There were no significant differences in age, sex, or Chalder FS scores measured before the intervention between the yoga group and the control group. The Chalder FS scores in the yoga group decreased significantly from 25.9 ± 6.1 pre-intervention to 19.2 ± 7.5 after the 2-month yoga intervention (*P* = 0.002, paired-sample *t* test; Table 1), whereas scores did not change significantly in the control group (from 26.1 ± 6.2 to 25.8 ± 5.9). The Chalder FS scores were re-adopted from our previous study [25]. Two-way repeated ANOVA indicated that the groups differed in the change in Chalder FS scores (*P* = 0.004), with a large effect size (*d* = − 1.165) in favor of the CFS group (Table 2). Although there was a significant Time effect (*P* = 0.001) and Time x Group effect (*P* = 0.003), these effects did not differ between groups (*P* = 0.124). These findings suggest that CFS patients who practiced seated isometric yoga experienced a greater improvement in fatigue over time compared with those who did not practice yoga.

We compared the serum levels of cortisol, DHEA-S, TNF-α, IL-6, PRL, total carnitine, free carnitine, and acylcarnitine and the plasma levels of TGF-β1, BDNF, MHPG, HVA, and α-MSH before versus after intervention in the yoga group (Table 1). However, 2 months of practicing seated isometric yoga did not significantly change any of the serum/plasma biomarkers evaluated.

We also compared ANS function, including the HR, HF, and LF components of HRV, LF/HF, and CV_R-R_ before versus after intervention in the yoga group. However, these parameters did not show significant changes over the 2-month period (Table 1). The basal respiration rate also showed no change.

### Longitudinal effects of yoga on psychological parameters

We assessed the effects of seated isometric yoga on depression, anxiety and alexithymia by comparing the HADS-D, HADS-A, and TAS-20 scores before versus after intervention. These values were also compared between the yoga group and the control group. Before the intervention, HADS-D, HAD-A, and TAS-20 scores did not differ between the yoga group and the control group. In the control group, HADS-D, HAD-A, and TAS-20 scores did not change significantly after the intervention period: the HADS-D score changed from 9.3 ± 3.5 to 10.1 ± 3.8 (not significant), HADS-A changed from 9.5 ± 3.8 to 9.0 ± 3.2 (not significant), and TAS-20 changed from 56.1 ± 8.1 to 55.9 ± 7.8 (not significant). In the yoga group, however, the HADS-D score was decreased significantly (from 8.3 ± 4.6 to 5.7 ± 5.4, *P* = 0.02) after the intervention, whereas the HADS-A and TAS-20 scores did not show a significant change (Table 2). At the time of the post-intervention evaluation, the HADS-D score of the yoga group was significantly lower than that of the control group (*P* = 0.014), suggesting that patients who practiced yoga for 2 months experienced greater improvement in depressive symptoms than those who did not. Two-way repeated ANOVA indicated that the groups differed in the change in their HADS-D scores (*P* = 0.008, independent-sample *t* test), with a large effect size (*d* = − 1.062) in favor of the CFS group (Table 2). There was a significant Time x Group effect (*P* = 0.008), but this effect did not differ between the groups (*P* = 0.075). These findings suggest that CFS patients who practiced seated isometric yoga experienced a greater improvement in depression, regardless of the time effect, compared to those who did not practice yoga. There were no significant differences in HADS-A and TAS-20 scores between the groups (Table 2).

### Correlations between the change in fatigue level and changes in psychoneuroimmunological parameters in the yoga group

**Fig. 2:**
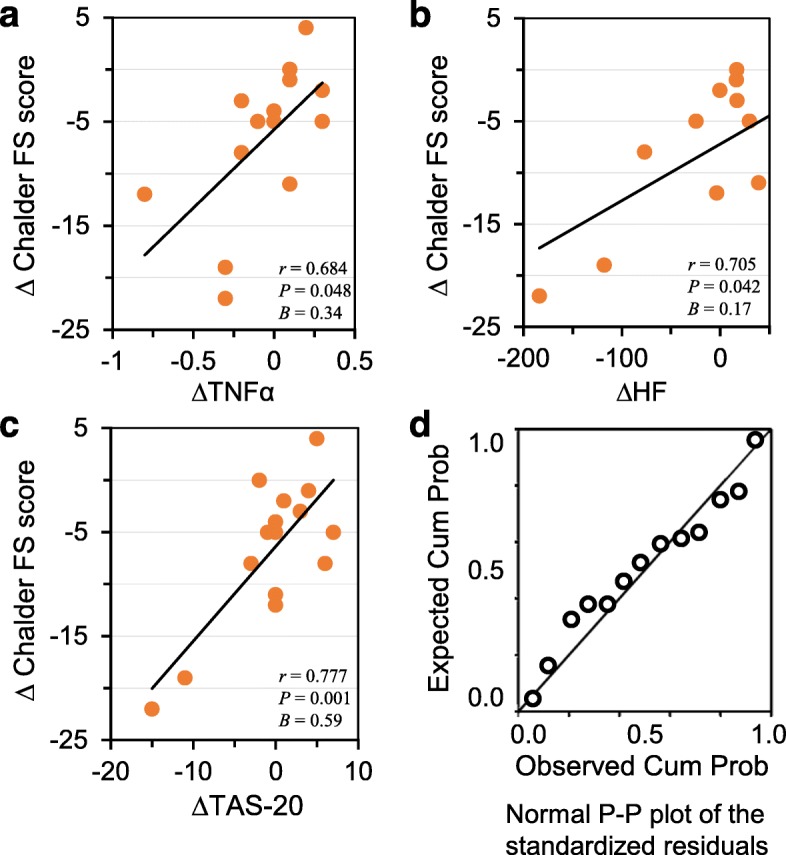
Correlations between the ∆Chalder FS score and ∆TNF-α (**a**), ∆HF (**b**), and ∆TAS-20 scores (**c**); each trend line indicates a linear relation between two respective variables. The normal P-P plot of the standardized residuals of the multiple regression analysis (**d**): ∆Chalder FS score as the dependent variable, ∆TNF-α, ∆HF, and ∆TAS-20 as the independent variables. This indicates that the residuals are normally distributed, and there are no outliers or influential points

We next assessed the correlation between the changes in Chalder FS scores and the changes in the other parameters. Pearson’s bivariate correlation analysis showed that the change in the Chalder FS score (mean: − 6.7 ± 6.9) was not correlated with the change in the HADS-D score (Table 2). In contrast, Chalder FS scores were positively correlated with the changes in TNF-α levels (mean: − 0.1 ± 0.3, *r* = 0.684, *P* = 0.048; Fig. 2a), the HF component of HRV (mean: − 4.3 ± 87.4, *r* = 0.705, *P* = 0.042; Fig. 2b), and the TAS-20 score (mean: − 0.4 ± 5.9, *r* = 0.777, *P* = 0.001; Fig. 2c). Furthermore, a multiple linear regression analysis shows that 80% of the variance of ∆ Chalder FS score (*intercept* = − 5.9, *F*_(3)_ = 11.89, *P* = 0.002) was explained by ∆TNFα (*B* = 0.34), ∆HF (*B* = 0.17), and ∆TAS-20 (*B* = 0.59). A normal P–P plot of the standardized residuals indicated a normal distribution of the residuals with no strong deviations (Fig. 2d). None of the variables broke the test of normality by the Kolmogorov-Smirnov test, and the assumption of homoscedasticity was met. There was no first order linear auto-correlation by the Durbin-Watson test (d = 2.17) and no multicollinearity by VIF values (each value < 10).

## Discussion

This pilot study demonstrated that regular practice of seated isometric yoga for 2 months significantly reduced Chalder FS and HADS-D scores. A decrease in the Chalder FS score was positively correlated with reductions in serum TNF-α levels, the HF component of HRV, and the TAS-20 score. These findings suggest that longitudinal practice of seated isometric yoga alleviates the depression of patients with CFS. This study also demonstrated that the changes in Chalder FS scores were associated with changes in blood biomarkers (TNF-α), ANS function (HF), and psychological parameters (alexithymia), although these parameters did not reach a significant reduction after practicing yoga. To our knowledge, this is the first study to investigate the longitudinal effects of isometric yoga on blood parameters, ANS function, and psychological parameters of patients with CFS.

### HPA axis parameters

Several studies have suggested the existence of HPA axis dysfunction in patients with CFS, including attenuated diurnal changes in cortisol levels [4, 5] and decreased DHEA-S levels in the blood [6, 7]. The pathophysiological effect of HPA axis hypofunction on CFS patients is still unclear. One possibility is that hypofunctional of the HPA axis may lead to excessive proinflammatory cytokine release, e.g. IL-6 and TNF-α, which causes symptoms such as flu-like malaise [50]. In our previous study, we demonstrated that a single session of seated isometric yoga significantly decreased the serum cortisol levels, suggesting short-term anti-stress effects [26]. Furthermore, it also increased serum levels of DHEA-S [26], which buffers the negative effects of stress [51]. As stress exacerbates CFS symptoms [3, 33], both changes may be beneficial for CFS patients. Regular practice of seated isometric yoga, however, did not affect these values when assessed in the afternoon.

From this study, we cannot determine if regular practice of seated isometric yoga can restore the blunted rhythm of cortisol secretion for patients with CFS. Previous studies have demonstrated that regular practice of yoga improved the diurnal cortisol rhythm of cancer patients [27] and that it increased the cortisol awakening response of healthy subjects [52]. Therefore, it is promising and worthy to investigate if this may also be the case in patients with CFS.

### Immune function parameters

Proinflammatory cytokines, e.g. IL-6 and TNF-α, induce sickness behavior, such as decreased spontaneous activity and sickness-associated physical symptoms such as fever and hyperalgesia [53–56]. As these symptoms resemble those that patients with CFS manifest, numerous studies have investigated the involvement of cytokines in CFS. Although the findings are still inconsistent [57], several studies have shown that blood levels of several cytokines are higher in patients with CFS than healthy subjects; these include TNF-α, IL-6, and TGF-β1 [8–11, 58, 59].

In our previous study, a single session of isometric yoga decreased serum levels of TNF-α [26]. Although regular practice of seated isometric yoga did not decrease serum TNF-α significantly, this study demonstrated a positive correlation between TNF-α levels and the Chalder FS score. Another study also reported positive correlations between TNF-α levels and the severity of sadness, autonomic symptoms, and the flu-like malaise of patients with CFS [9]. Therefore, TNF-α may play a role in the symptom-relieving effects of seated isometric yoga.

In contrast, TGF-β1 has both pro- and anti-inflammatory properties [59]. Our previous study showed that a reduction of TGF-β1 levels in the blood was associated with a seated isometric yoga-induced short-term reduction of fatigue [26]. However, such association was not found with the longitudinal reduction in Chalder FS scores. The reasons for this discrepancy are unclear. However, the role of TGF-β1 might be limited compared to that of TNF-α.

BDNF has been associated with improvements in depression, which is one of the common comorbid psychiatric disorders in CFS [23]. Given that regular yoga practice has been reported to increase the blood levels of BDNF of healthy subjects [60] and chronic pain patients [61], we anticipated that regular practice of seated isometric yoga could also increase the BDNF of patients with CFS. However, this study did not show significant changes in BDNF resulting from regular seated isometric yoga.

### CNS parameters

Several studies have suggested dysfunction of the dopaminergic [12, 13] and noradrenergic nervous systems [16] in the CNS of CFS patients. For example, patients with CFS are reported to exhibit reduced neural activation in the caudate nucleus in response to a reward task [12] and in the putamen [13], which may be due, in part, to dopaminergic dysfunction [12, 13]. To assess dopaminergic function, we measured changes in the plasma levels of HVA and serum levels of PRL. HVA is a major metabolite of dopamine reported to reflect dopaminergic neural tone [14, 15, 62] and dopamine acts as a PRL-inhibiting hormone [63]. Our previous study demonstrated that a change in HVA levels was positively correlated with a change in the vigor score on the Profile of Mood States [26], which suggests that dopaminergic activation is related to improvement of energy. Furthermore, a single session of seated isometric yoga had a tendency to reduce PRL serum levels, suggesting activation of the dopaminergic system. Therefore, these effects may be beneficial for patients with CFS. However, the changes induced by a single session of isometric yoga may be short-lived and transient as regular practice of seated isometric yoga failed to induce such effects.

Plasma levels of MHPG, a major metabolite of noradrenaline [14, 15], are reported to be lower in patients with CFS than in healthy subjects [16]. Neither a single session, which was reported in our previous study, nor regular practice, as shown in the present study, of seated isometric yoga induced any changes in the plasma levels of MHPG. These results suggest that seated isometric yoga may not affect the noradrenergic system.

The plasma α-MSH of CFS patients is reported to be higher than that of healthy subjects [17]. α-MSH is released from the melanotroph of the intermediate lobe of the pituitary gland and exerts anti-inflammatory effects [53, 54]. An animal study suggested that fatigue-inducing prolonged stress suppresses hypothalamus-derived dopamine release in the intermediate lobe and elicits hyper-secretion of α-MSH [64]. Therefore, considering the stress-reducing effects of isometric yoga, it is reasonable to hypothesize that isometric yoga may reduce plasma levels of α-MSH. However, the present study showed that this was not the case.

### ANS function parameters

Several studies have demonstrated that, compared with healthy subjects, patients with CFS have a higher resting HR [18–20] and lower cardiac vagal indices as assessed by HRV [20, 65, 66]. In our previous study, a single session of yoga increased the HF component of HRV, an indicator of cardiac vagal tone [26]. Therefore, the short-term changes in ANS function induced by seated isometric yoga seem to be beneficial and therapeutic for patients with CFS. In the present study, we hypothesized that regular yoga practice would also decrease HR and increase the HF component of HRV and that these changes would be associated with a yoga-induced decrease in fatigue. However, yoga practice did not affect any of the parameters evaluated. In contrast, the change in Chalder FS scores showed an association with the change in the HF component of HRV. This finding suggests that a longitudinal reduction in fatigue is associated with decreased vagal tone. This result was not in line with our expectations, and the reasons are uncertain. One possible explanation might be that some patients who had been nearly bed-bound previously were able to remain awake during the daytime and begin working again after an improvement in their fatigue following the yoga intervention. HF becomes lower when patients with CFS are awake compared to when they are asleep [66–68]. Improved fatigue might lead to increased daily activity and thus a decrease in vagal activity. Further studies will be necessary to test this hypothesis.

### Metabolic parameters

Carnitine has an important role in energy production and modulation of the intramitochondrial coenzyme A (CoA)/acyl-CoA ratio in skeletal muscle. Carnitine deficiency causes abnormal energy metabolism, resulting in muscle weakness or myalgia [69, 70], and thus the involvement of carnitine in CFS was investigated. Previous studies demonstrated that serum acylcarnitine, but not free carnitine, was lower in patients with CFS than healthy subjects and that the acylcarnitine concentration recovered as CFS symptoms improved [21, 22]. The present study did not show any changes in serum levels of either free or acylcarnitine by regular practice of seated isometric yoga. Therefore, changes in acylcarnitine may not account for the longitudinal, fatigue-relieving effects of this type of yoga intervention.

### Psychological indices

Patients with CFS sometimes suffer from comorbid reactive depression [23], and thus show higher HADS-D scores than healthy subjects [24, 71, 72]. In this study, we assessed the effects of regular practice of isometric yoga on HADS-D (depressive symptoms), HADS-A (anxiety), and TAS-20 (alexithymia) and found that isometric yoga exclusively improved HADS-D scores, suggesting an improvement in depressive symptoms by regular practice of yoga. As comorbid depression has been demonstrated to increase the level of psychophysical distress in patients with CFS [73], the seated isometric yoga-induced reduction in depressive symptoms must be beneficial for them.

This study also demonstrated that changes in the Chalder FS score were associated with changes in the TAS-20 score, suggesting that a longitudinal improvement in fatigue is associated with reduced alexithymia. A previous study reported higher TAS-20 scores for patients with CFS than for healthy subjects [24]. In accordance with this study, the TAS-20 score of patients with CFS (55 ± 13) in this study was higher than of a healthy Japanese sample (48 ± 9) [74]. To date, the pathophysiological significance of alexithymia in CFS is not fully understood. A functional magnetic resonance imaging study demonstrated greater activation of the prefrontal brain regions, which are implicated in emotional regulation, during anxiety-provoking conditions in patients with CFS compared with healthy controls [72]. This suggests that CFS patients tend to over-regulate or suppress their emotional response to anxiety-provoking stimuli [72], which might be related to the higher TAS-20 score of these patients. Conversely, the isometric yoga-induced improvement in alexithymia is potentially associated with improved regulation of this emotional response. This is the first study to suggest an association between improved fatigue and improved alexithymia. Based on these findings, future treatment of alexithymia might provide a possible strategy for treating CFS [75].

#### Limitations

This study has several limitations. First, the sample size was relatively small because the subjects were the same as those enrolled in the yoga group of our previous RCT [25] and because there were insufficient blood samples to measure all blood biomarkers. Second, all patients enrolled in this study were capable of sitting for at least 30 min; therefore, it is unclear whether the present findings can be generalized to patients with more severe CFS. Third, autonomic function testing and blood sampling were conducted at one time point, between 2 p.m. and 4 p.m., both pre- and post-intervention. Therefore, we might have missed some valuable changes that could be evident if measured at multiple time points. Because of this limitation, we could not determine whether yoga had any effects on diurnal changes in the HPA axis, especially the cortisol awakening response. However, this limitation could not be avoided given the condition of some patients. For example, some patients were unable to wake up and visit the hospital in the morning. Furthermore, it took some of them several hours to reach the hospital from their homes. As this study was conducted in an outpatient setting, it was most feasible to see patients and obtain samples in the afternoon, when their conditions were relatively stable and when diurnal changes in some parameters, e.g. cortisol and HRV, and the effect of diet on parameters were minimal. Fourth, this study did not include a follow-up assessment. This was due to a high level of satisfaction among the participants [25]. We initially planned for follow-up analysis at 16 weeks, i.e. 8 weeks after the intervention; however, 14 out of 15 participants asked to continue practicing isometric yoga, especially with an instructor, when they visited the hospital. Considering the efficacy of isometric yoga and the high satisfaction of the participants, we abandoned the follow-up analysis in which patients would be asked to not practice isometric yoga after the intervention period. To overcome these limitations, future studies will be necessary involving multiple sampling points and multiple assessment periods, which include a follow-up period and a larger sample size, including subjects with different illness durations or severity levels. Lastly, in spite of the significant improvement in fatigue, we failed to demonstrate any significant changes in blood biomarkers. Recent studies have suggested that microglial activation in the brain may play an important role in CFS [76]. Therefore, in the future, the effects of isometric yoga on neuroinflammation should be studied in addition to further analysis of the effects on other blood biomarkers.

Despite these limitations, to our knowledge, this is the first study to investigate the longitudinal effects of yoga on blood biomarkers, ANS function, and psychological parameters of patients with CFS. This study may provide some insights into our understandings on the role of yoga as an adjunctive therapy for CFS and its possible therapeutic mechanisms.

## Conclusions

This study demonstrated that regular practice of seated isometric yoga for 2 months reduced the fatigue and depressive symptoms of patients with CFS. Reduced fatigue severity was correlated with decreases in serum TNF-α levels, the HF component of HRV, and TAS-20 scores. These findings may provide insights into our understanding of the mechanisms underlying the longitudinal fatigue-relieving effects of seated isometric yoga for patients with CFS.

## Data Availability

Data sharing is not applicable.

## Abbreviations

A: Anxiety
ANS: Autonomic nervous system
BDNF: Brain-derived neurotrophic factor
CFS: Chronic fatigue syndrome
CV_R-R_: Coefficient of variation of R-R intervals
D: Depression
DHEA-S: Dehydroepiandrosterone sulfate
ELISA: Enzyme-linked immunosorbent assay
FS: Fatigue scale
HADS: Hospital anxiety and depression scale
HF: High frequency
HPA: Hypothalamic–pituitary–adrenocortical
HR: Heart rate
HRV: Heart rate variability
HVA: Homovanillic acid
IL: Interleukin
LF: Low frequency
ME: Myalgic encephalomyelitis
MHPG: 3-Methoxy-4-hydroxyphenylglycol
PRL: Prolactin
TAS-20: 20-item Toronto alexithymia scale
TGF: Transforming growth factor
TNF: Tumor necrosis factor
α-MSH: alpha-melanocyte stimulating hormone
